# Exogenously applied nitrogenous fertilizers and effective microorganisms improve plant growth of stevia (*Stevia rebaudiana* Bertoni) and soil fertility

**DOI:** 10.1186/s13568-021-01292-8

**Published:** 2021-09-27

**Authors:** Mohamed Ahmed Youssef, Ahmed Fathy Yousef, Muhammad Moaaz Ali, Alshaymaa I. Ahmed, Sobhi F. Lamlom, Wacław Roman Strobel, Hazem M. Kalaji

**Affiliations:** 1 Department of Soils and Water Sciences, Faculty of Agriculture, Al-Azhar University (Assiut Branch), Assiut, 71524 Egypt; 2 Department of Horticulture, College of Agriculture, University of Al-Azhar (Branch Assiut), Assiut, 71524 Egypt; 3 College of Horticulture, Fujian Agriculture and Forestry University, Fuzhou, 350002 China; 4 Agriculture Microbiology Department, Faculty of Agriculture, Beni-Suef University, Beni-Suef, Egypt; 5 Plant Production Department, Faculty of Agriculture Saba Basha, Alexandria University, Alexandria, 21531 Egypt; 6 Institute of Technology and Life Sciences, Falenty, Al. Hrabska 3, 05-090 Raszyn, Poland; 7 Department of Plant Physiology, Institute of Biology, Warsaw, University of Life Sciences SGGW, 159 Nowoursynowska 159, 02-776 Warsaw, Poland

**Keywords:** Stevioside, Nitrogen recovery efficiency (NRE), HPLC, Soybean protein isolate, Biofertilizer

## Abstract

The effects of different fertilizers and biofertilizers on crop production to increase plant growth, improve quality and yield components (dry leaves yield, leaf protein, and stevioside) of crops has been extensively studied. However, the combination of both types of fertilizers have rarely been investigated. To explore the effect of different fertilizers and biofertilizers on stevia plant, a two-year field experiment was conducted to investigate the growth response of stevia plants under the influence of nitrogenous fertilizers (NFs) and effective microorganisms (EM). The experiment was laid out in a split-plot design, with EM as the main plot factor (−EM and +EM) and NFs as the subplot factor [control, chemical NFs (Ch-N) and organic NFs (Org-N)]. The results showed that, plants treated with EM and Org-N showed 2-, 2.2-, 2.4-, 2.5-, 3.3- and 3-fold increases in plant height, number of branches, total leaf area, plant fresh weight, plant dry weight and leaf dry yield, respectively, compared to untreated plants. Similarly, plants receiving EM along with Ch-N showed 1.86-, 1.7-, 2.2-, 2.12-, 3-, and 2.72-fold increases in the same traits. Total chlorophyll, protein, N, P, K and sativoside contents were increased by 88.8, 152, 138, 151.5, 43 and 137.5% when EM and Org-N were applied to stevia plants. Application of EM together with Ch-N increased these properties by 0.5, 127.7, 115, 216, 42.6 and 83.8%, respectively in the same traits. Overall, the combined application of NFs and EM improved growth, yield and nutrient accumulation in stevia plants.

## Introduction

Stevia (*Stevia rebaudiana* Bertoni) is one of nearly 300 plant species belonging to the genus Stevia present all over the world, is known as the sweetest plant, sweet leaf, honey yerba, etc. It was originated from South America, officially discovered by Bertoni in 1905, belonging to the Asteraceae family (Das et al. 2007; Zaman et al. 2015b). Currently, the plant is cultivated in several countries including Japan, China, India, Korea, Taiwan, Philippines, Russia, Tanzania, Indonesia, Hawaii, Canada, Malaysia and Egypt (Rios and Recio 2005). It has become a popular natural source of high potency sweetener, dietary supplement and pharmaceutical products. For this reason, it has a significant impact on the economy of many countries (Debnath 2007; Huda et al. 2007; Zaman et al. 2015c).

The sweetness obtained from Stevia is 100 to 300 times sweeter than sucrose sugar which is extracted from sugar cane and sugar beet (Lemus-Mondaca et al. 2012). It has numerous health benefits against diabetes, hypertension, obesity, cancer, oxidative stress, and microbial infections (Ghanta et al. 2007). It is safe for diabetics and hypoglycemics being a healthy and natural sweetener (Ahmad et al. 2020; Kumar et al. 2013). Therefore, stevia represents a crop gaining much popularity among all types of sweeteners as a source of intensively sweet-tasting compounds, i.e., the steviol-glycosides. Stevia leaves contain steviol-glycosides mainly stevioside, rebaudioside A, rebaudioside B, rebaudioside C, and rebaudioside E, etc., and has not been observed any mutagenic, teratogenic, or carcinogenic effects, indicating it as an ideal substitute of sugar (Pól et al. 2007). Stevioside is one of the active constituents that have the largest share in stevia leaves (5–10% of dry weight basis) (Chumthong and Detpiratmongkol 2016; Das et al. 2008; Patil 2010).

Recently, researchers have been reported on using alternative nutrients to improve plant growth, development, and productivity under various environmental stresses. Elsheery et al. (2020a) have recommended that a combination of 100 mg L^−1^ nano-zinc oxide (nZnO) and 150 mg L^−1^ d nano-silicon (nSi) improves mango tree resistance, annual crop load, and fruit quality under salinity conditions. It has been recommended that a combination of 0.30% humic acid + 600 mg·L^−1^ boric acid to enhance the withstand environmental stresses and improve annual mango tree productivity and fruit quality (El-Hoseiny et al. 2020). The silicon dioxide (nSiO) showed higher amelioration effects and it can be used alone or in combination with other nanoribbons (zinc oxide, selenium, and graphene) to mitigate chilling stress in sugarcane (Elsheery et al. 2020b).

Stevia, being a vegetatively productive plant mainly needs nitrogen supplementation (Hasnain et al. 2020). Nitrogen is considered as an essential nutrient for foliage growth of plants (Hardjowigeno 2007). It is primarily available in the form of NO_3_^−^ and NH_4_^+^, and plants show a strong preference for NO_3_^−^ over NH_4_^+^ ions (Zhou et al. 2011). Sufficient nitrogen in the soil plays a vital role in the formation of chlorophyll for the photosynthesis (Tadesse 2019). Chemical nitrogenous fertilizers are one of the main factors accelerating global warming that may cause some environmental problems, such as harmful algal bloom, loss of aquatic life and increasing gas nitrous oxides (Savci 2012; Sedlacek et al. 2020). Though, most of the organic nitrogenous fertilizers are more available and eco-friendly, it is necessary to opt them as an alternative source of the chemical nitrogen used in plant nutrition (Bulluck Iii and Ristaino 2002; Naser et al. 2016).

The proper and judicious use of organic nitrogen fertilizer has become essential to meet the nutritional demand of plants and preserve the environment (Leghari et al. 2016). Using biofertilizers is one of the most critical steps in crop production to increase plant growth, improve fruit quality and yield components of crops through the way of various biochemical activities (Yakhin et al. 2017). In recent years, the investigations proved ‘soybean protein’ as an excellent source of organic matter for plants, especially being rich in nitrogen contents (Anbu and Saranraj 2016). Along with soybean protein, effective microorganisms (EM) can improve soil fertility status (Chumthong and Detpiratmongkol 2016; Das et al. 2008; El-Sirafy et al. 2015; Kumar et al. 2013; Liu et al. 2011; Shalini and Gupta 2010; Yang et al. 2013). EM can increase the availability of soil nutrients through their biological activity and nutrients uptake by plants (Abdel-Gawad and Youssef 2019; Bargaz et al. 2018; Das et al. 2007, 2008; Meena et al. 2014; Rajasekaran et al. 2015; Saraswati and Sumarno 2008).

Hence, in current study, we not only investigated the individual and combined effect of EM and nitrogenous fertilizers on yet unexplored aspects of stevia plant growth but also segregated treatment-dependent variations in plant vegetative growth, yield, and nutritional attributes of stevia.

The aim of this study was to explore how effective microorganisms and nitrogenous fertilizers differentially regulated various aspects of plant growth and development of stevia plants.

## Material and methods

### Experimental site, climate and soil

**Fig. 1:**
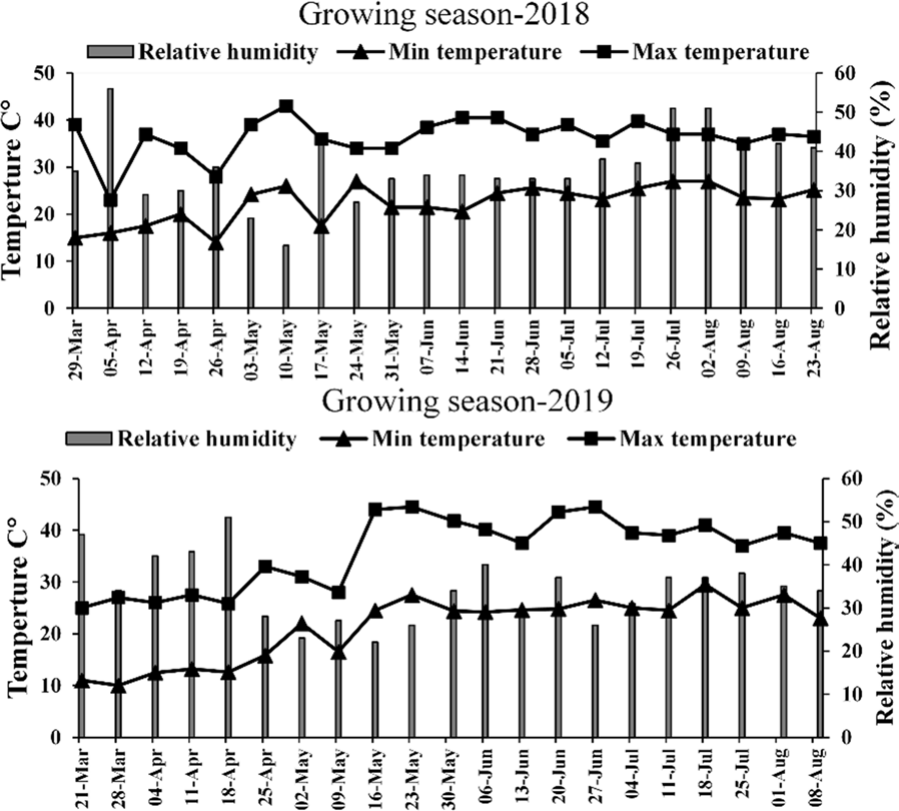
Weather conditions during the two growing periods of Stevia cultivation

A 2-years field experiment was carried out at research station of the “Soils and Water Department, Faculty of Agriculture, Al-Azhar University, (Assuit branch), Assuit governorate, Egypt” (27° 12ʹ 16.67ʺ N; 31° 09ʹ 36.86ʺ E). The climate of this area is characterized as very hot and dry in summer and cold in winter (BWh according to Köppen–Geiger climate map) (Beck et al. 2018) (Fig. 1). The initial physicochemical properties of the soil were determined during both seasons according to Carter (1993) (Table 1). Before preparing the soil for cultivation, soil samples were collected from each plot at one depths (0–30 cm) using a spiral auger of 2.5 cm diameter. Three sub-samples from each plot were taken to make a composite soil sample per treatment. They were transported to the laboratory, oven-dried at 40 °C and crushed to pass through a 2 mm sieve, and then ground to < 60 µm for determination of soil organic carbon content (SOC%), N, P-available (mg kg^−1^), and K-exchangeable (cmol kg^−1^) (Madejón et al. 2006). Furthermore, electric conductivity (E.C.) (µS cm^−1^) and soil pH were estimated through standard procedures as described by Carter (1993).

**Table 1:** Some physical and chemical characteristics of soil experimental

Parameters	Values	
	2018	2019
Particle size distribution		
Sand (%)	46.30	49.25
Silt (%)	28.40	30.43
Clay (%)	25.30	20.32
Texture grade	Silt clay loam	Silt loam
Available nutrients (mg kg^−1^)		
N	77.48	89.28
P	30.35	35.84
K	379.32	436.53
Soluble cations and anions (cmol kg soil)		
Ca^++^	0.88	0.94
Mg^++^	0.69	0.75
Na^+^	0.43	0.58
K^+^	0.32	0.47
HCO_3_^−^	0.67	0.73
Cl^−^	0.53	0.66
SO_4_^−−^	0.41	0.57
Chemical properties		
pH (Susp. 1:2.5 soil–water)	8.23	7.96
EC_e_ (dSm^−1^)	0.686	0.786
C.E.C (cmol kg^−1^)	18.76	19.38
Organic matter (%)	1.38	1.73
CaCO_3_ (%)	1.22	1.32

Each value in this table is the mean of 4 replicates

### Experimental design and treatments

This experiment was laid out in split-plot design having 2 levels of bio stimulant application as main plot [treated with EM (142.8 L ha^−1^) vs. untreated plants, hereafter EM+ and EM−, respectively], and 2 N fertilization types as sub-plot [chemical nitrogenous fertilizer (214 kg ha^−1^ N) and organic nitrogenous fertilizer (214 kg ha^−1^ N), hereafter Ch-N and Org-N, respectively] with four replicates. A N-unfertilized control was comparatively introduced in the experiment.

The soil was prepared by plowing twice orthogonally, then flattening the soil and dividing the experimental plots (4 × 3 m). Stevia seedlings were transplanted at a spacing of 30 × 50 cm (68 plants in each plot) under a drip irrigation system. The seedlings of *Stevia rebaudiana* Bertoni (Trade Name: Steviatech) were purchased from “Bio Tech Co., Giza, Egypt”. The seedlings were transplanted when 15–20 cm length was acquired (28th and 20th March in 2018 and 2019, respectively). The liquid Bio-fertilizer of EM containing *Lactobacillus casei* (9 × 10^7^ cfu), *Lactococcus lactis* (5 × 10^7^ cfu), *Saccharomices cerevisiae* (2 × 10^6^ cfu), *Rhodopseudomonas palustris* (4 × 10^6^ (cfu) including photosynthetic bacteria, lactic acid bacteria, and yeast [EMPO, Okinawa 901–2311, Japan (https://www.emrojapan.com)] was obtained from “Agricultural Research Center, Giza, Egypt”. Effective Microorganisms (EM) were applied 30, 51, 72, 93, 114, and 135 days after transplanting (DAT) with immersed-way irrigation, the periods between the two waterings were range from 8–15 days (increased and decreased depending on the temperature). Ammonium nitrate having 33.5% N was used as the source of Ch-N and applied in three splits i.e., 30, 72, and 114 DAT. While isolated soybean protein having 15.36% N was applied as Org-N as basal dose. Hand weeding was carried out 48, 69, 90, and 110 DAT in both growing seasons. Chemical composition of isolated soybean protein was analyzed according to Motsara and Roy (2008) and listed in Table 2.

**Table 2:** Chemical composition of isolated soy protein samples used in the study on dry weight basis

Determination	values
N (%)	15.36
P (%)	1.25
K (%)	0.12
pH (Susp. 1:10)	6.46
EC dS/m (Ext. 1:10)	2.715
Organic C (%)	38.07
Organic matter (%)	65.64
C/N ratio	2.5:1
Bulk density (g/cm^3^)	0.36

Each value in this table is the mean of 4 replicates

### Data collection

Stevia plants were harvested in two cuttings (first cutting was performed 79 and 76 DAT, while second cuting 145 and 138 DAT in 2018 and 2019, respectively), at the stage when plants hold 10% of the flowers. Six plants from each plot were randomly choosen, and plant growth, yield and nutritional variables were recorded. The height of each plant was measured using meter rod from the soil surface to the top of the plant and number of branches were counted. The fresh weight of each plant was measured using digital weighing balance (MJ-W176P, Panasonic, Japan). Leaves from each plant were separated and weighed using the same digital weighing balance. Leaf area was estimated as described by Pandey and Singh (2011) For dry weight determination, plants were oven-dried at 65 °C until constant dry mass was attained (Sorgonà et al. 2011).

Before chemical analysis, the twenty leaves from the top were collected and washed three times with distilled water.. According to Motsara and Roy (2008), the nitrogen content of leaves was measured using the Kjeldahl apparatus. Phosphorus contents were determined using JENWAY 6305 UV/Visible Spectrophotometer at 643 nm (OD643) by the colorimeter method (ammonium molybdate) (Motsara and Roy 2008). Potassium contents in leaves were determined using a Flame Photometer (BWB Model BWB-XP, 5 Channel) as described by Motsara and Roy (2008). Protein content in leaves (expressed on a percentage basis) was calculated as N content (%) × 6.25 (Mariotti et al. 2008). Chlorophyll contents from fully developed leaves were determined using a mobile chlorophyll meter (SPAD-502-m Konica Minolta, Inc., Tokyo, Japan). The performance of the chlorophyll meter was calibrated according to the manufacturer’s instructions, before taking the readings. The stevioside contents were estimated through High-Performance Liquid Chromatography (HPLC) following the protocol of Nishiyama et al. (1992).

### Statistical analysis

Collected data were subjected to a split-plot analysis of variance (ANOVA) using SPSS statistical software package version 16.0 (SPSS Inc., Chicago, IL, USA). Significant differences between treatment means were executed using Fisher’s least significant difference (LSD) test, when level of significance was 5% (*p* ≤ *0.05*).

## Results

### Plant height, number of branches, leaf area, plant fresh weight, plant dry weight and dry leaves yield

In first season (2018), the plants receiving EM exhibited better plant height (48.55 cm), number of branches per plant (34.44), leaves area per plant (1093.64 cm^2^), plant fresh weight (93.75 g), plant dry weight (24.69 g), and leaf dry yield (669.23 kg ha^−1^) as compared to untreated plants. Among nitrogenous fertilizers treatments, the plants treated with Org-N showed better plant height (56.58 cm), number of branches per plant (41.83), plant fresh weight (117.22 g), plant dry weight (31.43 g), leaves area per plant (1305.9 cm^2^) and dry leaves yield (859.58 kg ha^−1^) as compared to untreated plants and those treated with Ch-N. Considering the combined effect of EM and NFs, the highest values of same attributes (59.50 cm, 43.33 plant^−1^, 119.86 g, 32.96 g, 1335.27 cm^2^ and 17.39 g, respectively) were recorded in the plants provided with Org-N along with EM. Similarly, in second growing season (2019), the plants receiving EM exhibited 1.09, 1.09, 1.04, 1.09, 1.14, and 1.11-fold increase in plant height, number of branches, leaves area per plant, plant fresh weight, plant dry weight, and leaves dry yield as compared to untreated plants, respectively. Among nitrogenous fertilizers treatments, the plants treated with Org-N, being best treatment showed 2.04, 2.28, 1.97, 2.13, 3.14, and 3.05-fold increase in same attributes as compared to untreated plants and those treated with Ch-N. Considering the combined effect of EM and NFs, the plant height, number of branches, leaves area per plant, plant fresh weight, plant dry weight, and leaves dry yield were increased by 112.45, 152.97, 110.88, 142.74, 265.38 and 243.60%, respectively, when EM and Org-N were applied to stevia plants. While, by the application of EM along with Ch-N, these attributes were enhanced by 93.77, 88, 99, 88.77, 226.73 and 209.87%, respectively (Tables 3, 4).

**Table 3:** Effect of nitrogenous fertilizers and effective microorganism on plant height, number of branches and leaf area of *Stevia rebaudiana* during two growing seasons

Growing season	Treatments	Plant height (cm)			No of branches			Leaf area (cm^2^ plant^−1^)		
		EM (−)	EM (+)	Mean (NFs)	EM (−)	EM (+)	Mean (NFs)	EM (−)	EM (+)	Mean (NFs)
2018	Control	29.72e	33.16d	31.44c	23.42d	24.16d	23.79c	498.5d	720.82c	609.66c
	Ch-N	46.50c	53.00b	49.75b	33.83c	35.83c	34.83b	1195.30b	1224.84ab	1210.07b
	Org-N	53.66b	59.50a	56.58a	40.33b	43.33a	41.83a	1276.53ab	1335.26a	1305.9a
	Mean (EM)	43.29b	48.55a		32.53b	34.44a		990.11b	1093.64a	
LSD interaction (*p* ≤ *0.05*)		2.47			2.89			126.42		
2019	Control	30.36d	31.33d	30.84c	25.43f	29.16e	27.3c	804.5c	900.4c	851.95c
	Ch-N	49.66c	58.83b	54.25b	44.16d	47.83c	46b	1571.68b	1601.22ab	1586.45b
	Org-N	61.33b	64.5a	62.91a	60.33b	64.33a	62.33a	1657.91ab	1696.54a	1677.23a
	Mean (EM)	47.12b	51.55a		43.31b	47.11a		1344.36a	1399.39a	
LSD interaction (*p* ≤ *0.05*)		2.62			1.814			124.79		

The values shown in table are means of two cuttings having four replicates. *EM* Effective microorganisms (biofertilizer), C*h-N* Chemical-N form, *Org-N* Organic-N form, Means followed by the same letters are non-significantly different (*p* ≤ *0.05*)

**Table 4:** Effect of nitrogenous fertilizers and effective microorganisms on plant fresh weight, plant dry weight and dry leaves yield of *Stevia rebaudiana* during two growing seasons

Growing season	Treatments	Plant fresh weight (g)			Plant dry weight (g)			Dry leaves yield (Kg ha^−1^)		
		EM (−)	EM (+)	Mean (NFs)	EM (−)	EM ( +)	Mean (NFs)	EM (−)	EM ( +)	Mean (NFs)
2018	Control	44.25e	56.85d	50.55c	10.86d	12.45d	11.65c	330.88d	337.62d	334.25c
	Ch-N	96.88c	104.56b	100.72b	25.64c	28.68b	27.16b	735.64c	776.08bc	755.86b
	Org-N	114.59a	119.86a	117.22a	29.9b	32.96a	31.43a	825.18ab	893.98a	859.58a
	Mean (EM)	85.24b	93.75a		22.13b	24.69a		630.57a	669.23a	
LSD Interaction (*P* ≤ *0.05*)		5.75			2.22			79.79		
2019	Control	60.13e	68.99d	64.56c	12.42d	14.62d	13.52c	355.41c	392.81c	374.11c
	Ch-N	110.96c	113.51c	112.23b	36.4c	40.58b	38.49b	1018.3b	1101.33b	1059.81b
	Org-N	129.05b	145.96a	137.51a	39.42bc	45.38a	42.4a	1063.37b	1221.19a	1142.28a
	Mean (EM)	100.05b	109.49a		29.41b	33.52a		812.36b	905.11a	
LSD Interaction (P ≤ *0.05*)		3.415			3.43			86.88		

The values shown in table are means of two cuttings having four replicates. *EM* Effective microorganisms (biofertilizer), *Ch*-*N* Chemical-N form, *Org-N* Organic-N form, Means followed by the same letters are non-significantly different (*p* ≤ *0.05*)

### Leaf nitrogen, phosphorus, and potassium

**Fig. 2:**
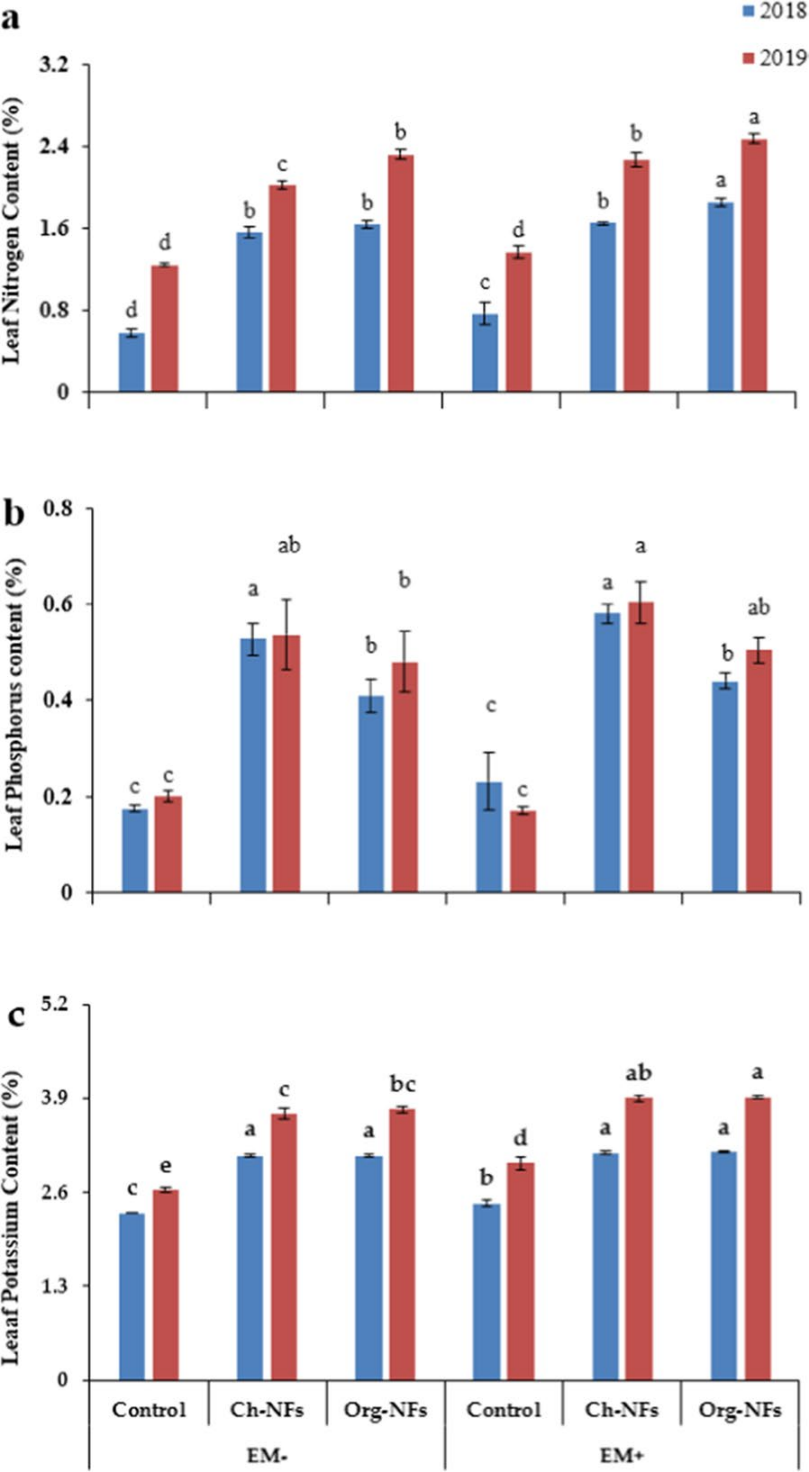
Effect of nitrogenous fertilizers and effective microorganisms on leaf nitrogen (**a**), phosphorus (**b**) and potassium (**c**) of stevia during two growing seasons (2018–19). *Ch*-*N* chemical nitrogenous fertilizer, *Org-N* organic nitrogenous fertilizer, *EM* effective microorganisms. Each value expressed in figure is mean of two cuttings. Vertical bars indicate mean ± standard deviation. Means followed by the same letter within the same series are not significantly different according to Fisher’s least significant difference (LSD) technique (p ≤ 0.05)

In both growing seasons, the maximum leaf nitrogen and potassium were recorded in the plants receiving Org-N along with EM, followed by the plants treated with Org-N without EM as compared to control. On the other hand, maximum phosphorus contents were recorded in the plants treated with Ch-N fertilizer, regardless of EM application. While the lowest average values for all observed nutritional attributes (N, P and K content) were observed in untreated plants during both growing seasons (Fig. 2).

### Leaf chlorophyll

**Fig. 3:**
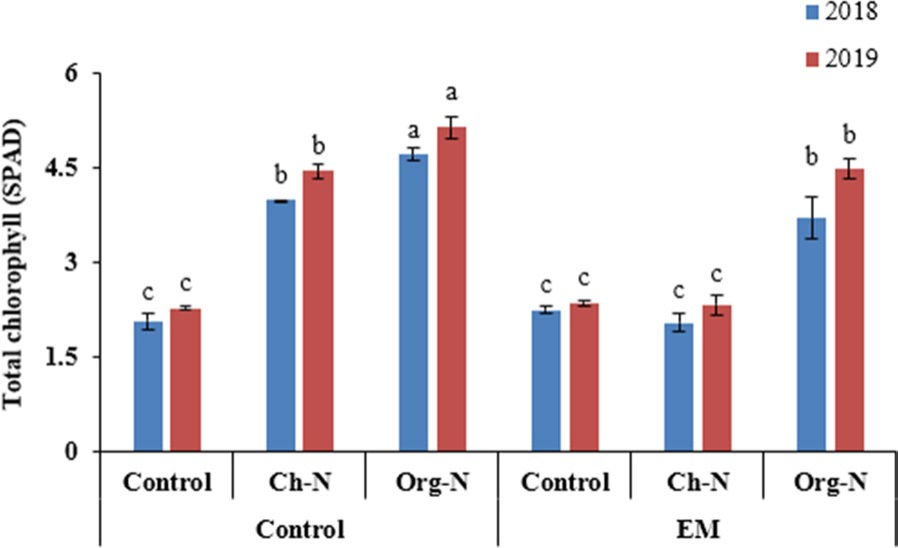
Effect of different nitrogenous fertilizers and effective microorganisms on leaf chlorophyll contents of stevia during two growing seasons (2018–19). *Ch*-*N* chemical nitrogenous fertilizer, *Org-N* organic nitrogenous fertilizer, *EM* effective microorganisms. Each value expressed in figure is mean of two cuttings. Vertical bars indicate mean ± standard deviation. Means followed by the same letter within the same series are not significantly different according to Fisher’s least significant difference (LSD) technique (p ≤ 0.05)

In both growing seasons, the plants treated with Org-N exhibited maximum leaf chlorophyll contents (4.72 and 5.14 mg g^−1^FW in 2018 and 2019, respectively) among all other treatments. Effective microorganisms could not influence the chlorophyll contents significantly (*p* ≤ *0.05*). Chemical nitrogen was also proved promising treatment after Org-N (Fig. 3).

### Leaf protein and stevioside content

**Fig. 4:**
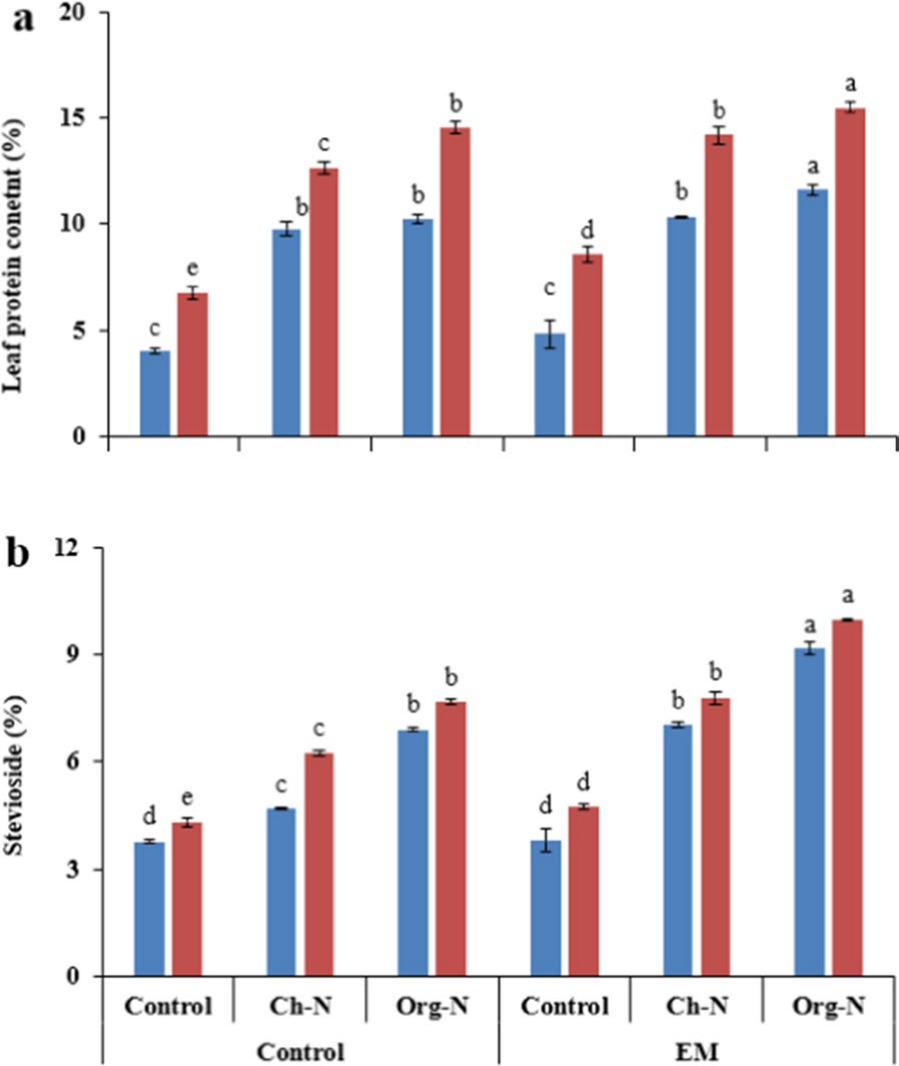
Effect of different nitrogenous fertilizers and effective microorganisms on leaf protein (**a**) and stevioside (**b**) contents of stevia during two growing seasons (2018–19). *Ch*-*N* chemical nitrogenous fertilizer, *Org-N* organic nitrogenous fertilizer, *EM* effective microorganisms. Each value expressed in figure is mean of two cuttings. Vertical bars indicate mean ± standard deviation. Means followed by the same letter within the same series are not significantly different according to Fisher’s least significant difference (LSD) technique (p ≤ 0.05)

During both growing seasons, maximum leaf protein contents (11.62 and 15.49% in 2018 and 2019, respectively) were recorded in the plants receiving Org-N along with EM. While the lowest values were observed in untreated plants during both growing seasons (Fig. 4a). Similarly, in case of leaf stevioside contents, the plants treated with the combination of Org-N and EM showed maximum performance (9.17 and 9.98% in 2018 and 2019, respectively). The EM uplifted the average stevioside contents by 23.52%, while Org-N enhanced the contents by 95.35%. On the other hand, the lowest stevioside contents (3.42 and 4.30% in 2018 and 2019, respectively) were recorded in untreated plants (Fig. 4b).

### Post-harvest fertility status of soil

**Fig. 5:**
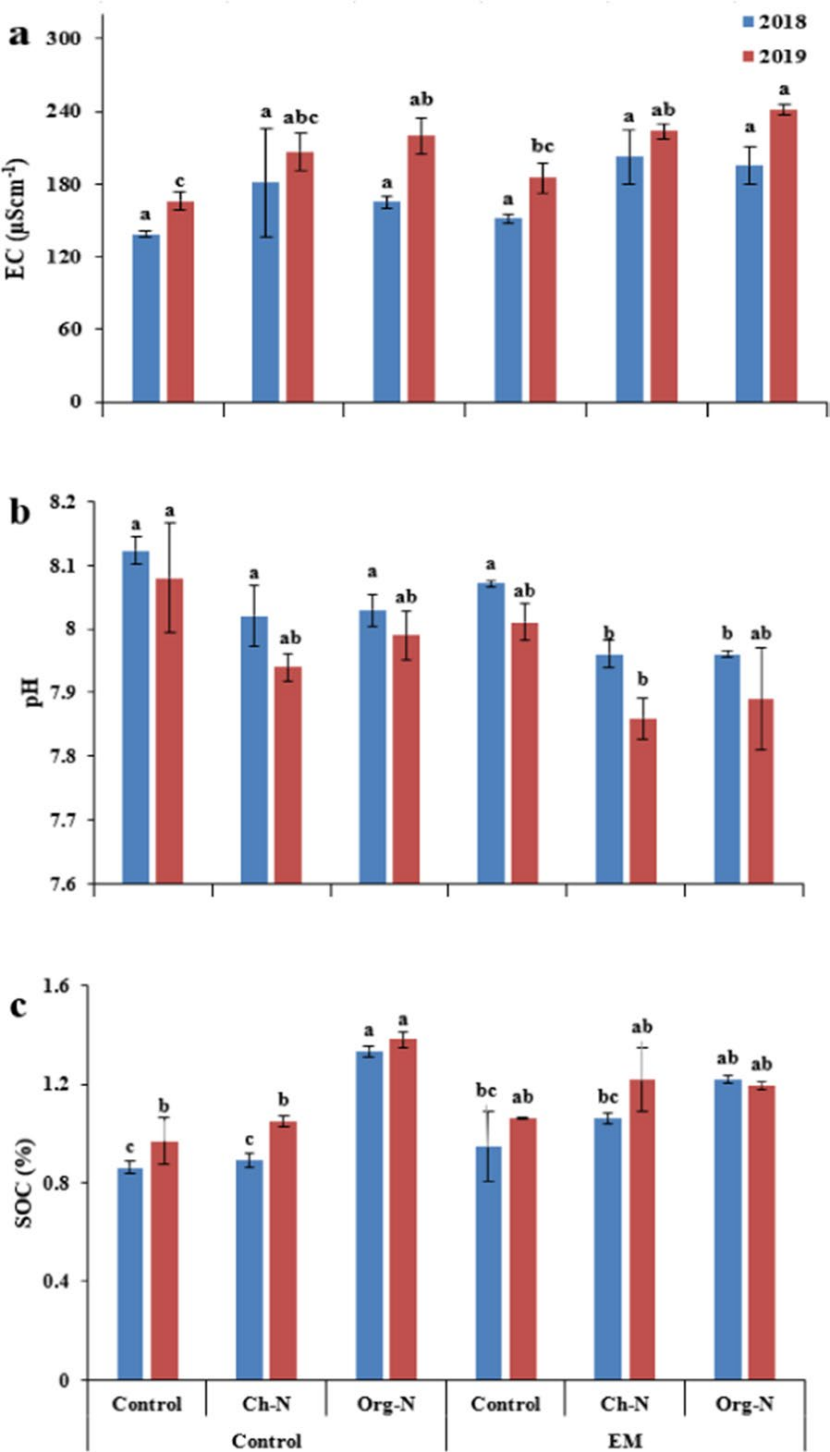
Effect of nitrogenous fertilizers and effective microorganisms on electric conductivity (a), pH (b), and soil organic carbon (c) of soil after harvest during two growing seasons (2018–19). *Ch-N* chemical nitrogenous fertilizer, *Org-N* organic nitrogenous fertilizer, *EM* effective microorganisms. Each value expressed in figure is mean of two cuttings. Vertical bars indicate mean ± standard deviation. Means followed by the same letter within the same series are not significantly different according to Fisher’s least significant difference (LSD) technique (p ≤ 0.05)

**Fig. 6:**
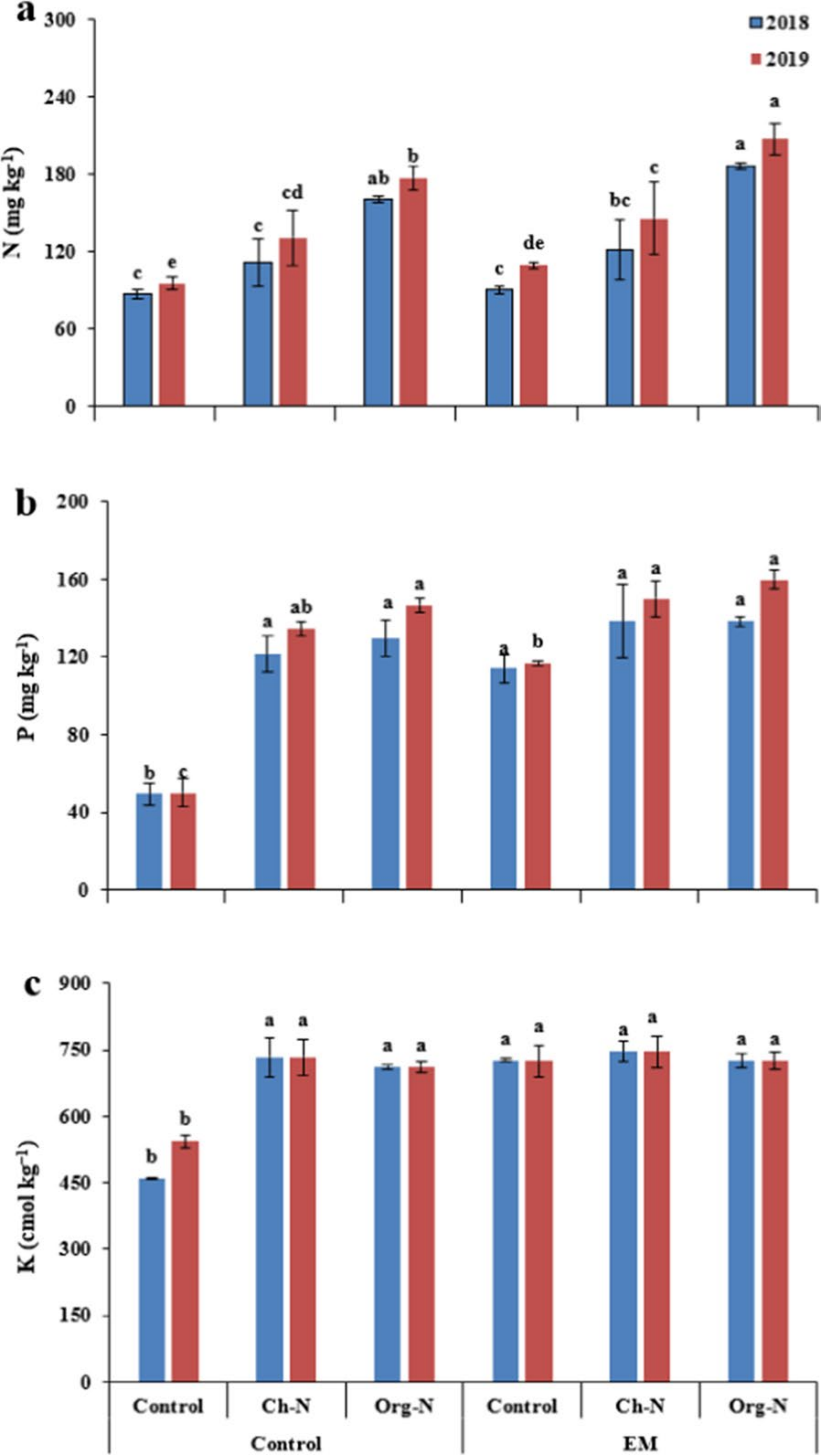
Effect of nitrogenous fertilizers and effective microorganisms on available nitrogen (**a**), phosphorus (**b**), and potassium (**c**) of soil after harvest during two growing seasons (2018–19). *Ch-N* chemical nitrogenous fertilizer, *Org-N* organic nitrogenous fertilizer, *EM* effective microorganisms. Each value expressed in figure is mean of two cuttings. Vertical bars indicate mean ± standard deviation. Means followed by the same letter within the same series are not significantly different according to Fisher’s least significant difference (LSD) technique (p ≤ 0.05)

The chemical properties of soil i.e., EC, soil pH, SOC, available N, P, and exchangeable K were significantly affected by the application of different nitrogenous fertilizers with or without effective microorganisms after stevia plantation (Figs. 5, 6). Under the influence of NFs, soil EC remained unchanged during 2018, while increased during 2019. Maximum EC was recorded as influenced by Org-N in 2019. Effective microorganisms significantly enhances soil EC during both growing seasons. Soil pH was reduced as affected by NFs in both growing seasons. Effective microorganism also played a significant role in reducing pH during 2019. The SOC increased by application NFs, while remained unchanged with EM application during both growing seasons (Fig. 5). During both growing seasons, NFs and EM individually and when applied in combination increased the contents of available N, P and exchangeable K in the soil (Fig. 6).

## Discussion

Nitrogen is the most difficult nutrient to manage in organic crop production (Aczel 2019). Cover crops and composts can provide a lot of N to plants, but synchronizing N release from these materials with plant demand is difficult (Mikkelsen and Hartz 2008). For example, soy protein isolate can be used as a nitrogen amendment that is degraded in the soil without pretreatment (Olbrycht et al. 2020). Olbrycht et al. (2020) showed that the proteins diffuse slowly through the soil and are degraded simultaneously, which is accompanied by the release of ammonium ions. In another study, grain number per ear, thousand-grain weight, total yield, soluble sugar, starch, fat, protein and vitamin C in maize were increased by the application of soybean protein hydrolysate modified with urea–formaldehyde fertilizer compared to urea–formaldehyde and control (Liu et al. 2019).

The positive effects of Org-N with EM on growth variables of stevia plants may be attributed to the efficiency of microorganisms of the biofertilizer in immobilizing N in the form of NH4^+^ for a longer period of time, which promoted nutrient uptake by the plant (Di and Cameron 2004). In a nursery experiment, Khan et al. (2006) discovered that EM had a positive effect on germination and seedling growth of Albizia saman in plants treated with different concentrations of EM solutions. Compared to untreated plants, EM treatments significantly increased vegetative growth, leaf chlorophyll content, and leaf mineral levels (N, P, K, Fe, Zn, and Mn) of apple trees (Cv. Anna) (Sahain et al. 2007). According to Olle et al. (2013) 70% of publications reported a positive effect of EM on vegetable growth. Therefore, reasonable use of Org-N with EM can improve the growth characteristics and yield of the stevia. Our results are in corroboration with various studies reporting, and the increased growth characteristics were achieved due to the addition of organic nitrogen combined with biofertilizer by Das et al. (2008) and Liu et al. (2011) in stevia plant. It has also been reported earlier that organic nitrogenous fertilizers enhance plant growth and improves the yield of stevia by Chumthong and Detpiratmongkol (2016), quinoa by Geren (2015) and wheat by Guo et al. (2014). In this concern, Mohamed et al. (2012) showed that the application of Org-N with EM increased leaf N, P, and K contents of stevia plants, indicating a positive role of nitrogen in forming chlorophyll for the photosynthetic process (Tadesse 2019).

For dry leaf yield of stevia, incorporation of Org-N with EM was superior to all other treatments (Table 4). The promoting effect of this treatment on dry leaf yield could be due to a positive interaction resulting from increased SOC, soil structure and available nutrients after cultivation (Oldfield et al. 2019; Sheng-zhe et al. 2018). Oldfield et al. (2019) found that yields of maize and wheat crops were higher on average when the concentration of SOC was higher. These results are also in agreement with the findings of Zaman et al. (2015a) and Rashwan et al. (2017) in stevia plant, and Youssef (2016) in moringa plant.

The Ch-N along with EM significantly increased the P, K and chlorophyll contents of stevia. The reason might be the positive interaction of the combined application of Ch-N and EM with improved properties of stevia. Yousef and co-workers discovered that the use of a combination of inorganic NPK and biofertilizer was most effective in enhancing growth, yield and nutrient accumulation in jew’s mallow plants (Yousef et al. 2020). Similar results were reported by Das et al. (2007), Das et al. (2008), Liu et al. (2011), Kumar et al. (2013), and Enchev et al. (2018) in stevia plant.

The results showed that the content of stevioside in stevia leaves was significantly increased with the increase in biomass yield by applying Org-N along with EM. Stevioside accumulation in stevia leaf is the “mirror” to stevioside yield, which was considered as the final goal for every researcher (Hamad 2015). The combined application of Org-N and EM improved the vegetative growth of stevia, net photosynthetic ability, so that the photosynthetic product increased the amount of SOC and improved soil structure. Moreover, Chumthong and Detpiratmongkol (2016) found that the application of organic manure was suitable to increase different growth parameters of stevia and stevioside content in stevia leaves. Rashwan et al. (2017) found that the application of compost at 4.76 ton ha^−1^ + 95 kg ha^−1^ N as NH_4_NO_3_ with 3rd cutting was the best treatment for improving the yield and quality of stevia.

In this study, it was found that the combination of EM with Org-N or Ch-N fertilizer resulted in a significant reduction in soil pH compared to either EM or Org-N or Ch-N treatment alone (Fig. 5). The difference in pH response to different fertilization regimes could be explained by one of two mechanisms. First, organic fertilization could have affected soil pH due to the liming effect of organic matter and carbonates of organic fertilizer (Cooper and Warman 1997). Second, ammonium can lower soil pH by completing the exchange sites of the soil solid phases with base cations (Li et al. 1991). In addition, different fertilization regimes altered soil nutrient status to varying degrees in this study. EM with combined Org-N or Ch-N fertilizer applications as well as Org-N alone significantly improved SOC. We can attribute the improved SOC with organic fertilizer treatments to two mechanisms. Unlike other treatments, extracted soy protein has a high concentration of organic compounds that were readily biodegradable (Yanardağ et al. 2015). These results are in agreement with Yousef et al. (2020), who reported that the application of organic manure and its combination with biofertilizer increased the SOC at the end of the experimental period. In addition, organic manure could promote plant growth, resulting in increased input of SOC into the soil through the plants (Ding et al. 2012).

The results of the present study showed that the growth, yield and chemical composition of stevia plants were significantly affected by different nitrogenous fertilizers and effective microorganisms. Plant height, number of branches, fresh weight, dry weight, leaf area, dry leaf yield and stevioside content of stevia were found maximum under the influence of combined application of organic nitrogen and effective microorganisms. Soil properties like pH, EC, SOC, available N, P and exchangeable K content were also greatly improved by nitrogenous fertilizers and effective microorganisms. Considering all the parameters and treatments studied, it can be concluded that the application of organic nitrogen along with effective microorganisms is a promising approach to produce higher yield and improve the quality of stevia. Although the current study unfolded the performance ability of an isolated soybean protein as organic nitrogenous fertilizer to improving the productivity of the stevia crop, there is further need to understand the molecular mechanism behind it and improve the fertilization techniques and material according to the need for crops.

## Data Availability

All data available within the article.
